# Relationship between metabolic reprogramming and drug resistance in breast cancer

**DOI:** 10.3389/fonc.2022.942064

**Published:** 2022-08-18

**Authors:** Linlin Lv, Shilei Yang, Yanna Zhu, Xiaohan Zhai, Shuai Li, Xufeng Tao, Deshi Dong

**Affiliations:** ^1^ Department of Pharmacy, First Affiliated Hospital of Dalian Medical University, Dalian, China; ^2^ School of Life Science and Biotechnology, Dalian University of Technology, Dalian, China

**Keywords:** Breast cancer, drug resistance, metabolic reprogramming, glucose metabolism, fatty acid synthesis

## Abstract

Breast cancer is the leading cause of cancer death in women. At present, chemotherapy is the main method to treat breast cancer in addition to surgery and radiotherapy, but the process of chemotherapy is often accompanied by the development of drug resistance, which leads to a reduction in drug efficacy. Furthermore, mounting evidence indicates that drug resistance is caused by dysregulated cellular metabolism, and metabolic reprogramming, including enhanced glucose metabolism, fatty acid synthesis and glutamine metabolic rates, is one of the hallmarks of cancer. Changes in metabolism have been considered one of the most important causes of resistance to treatment, and knowledge of the mechanisms involved will help in identifying potential treatment deficiencies. To improve women’s survival outcomes, it is vital to elucidate the relationship between metabolic reprogramming and drug resistance in breast cancer. This review analyzes and investigates the reprogramming of metabolism and resistance to breast cancer therapy, and the results offer promise for novel targeted and cell-based therapies.

## Introduction

Breast cancer is the primary cause of cancer-related death in women. The WHO reported approximately 2.26 million newly diagnosed cases of female breast cancer worldwide in 2020, which is equivalent to 1 in 8 cancer patients being breast cancer patients, rendering it the most common cancer in the world (1). According to GLOBOCAN data from 2020, China has the highest ASIR (age‐standardized rates of cancer incidence) of breast cancer, approximately 39.10 per 100,000 people (2). In 2019, GBD (Global Burden of Disease) data estimated breast cancer to be the main cause of the DALY (disability‐adjusted life year) burden in young and middle-aged women in the US and UK (3).

Currently, treatments for breast cancer are based on three broad classes: estrogen receptor α-positive or progesterone receptor-positive breast cancer, human epidermal growth factor receptor 2-enriched breast cancer, and triple-negative breast cancer that expresses none of these three receptors (4). Endocrine therapy can be effective for treating cancers in which either or both of the ER and PR proteins are overexpressed. Selective estrogen receptor modifiers (SERMs), aromatase inhibitors (AIs), and/or selective estrogen receptor degraders (SERDs) are among the endocrine therapies available. The subtype overexpressing HER2 was identified using HER2-targeted therapy. HER-2 targeted therapy can be achieved by monoclonal antibodies that are humanized, including trastuzumab and epratuzumab (5–7). Alternatively, HER2-positive patients may be treated with tyrosine kinase inhibitors, such as lapatinib (8) and neratinib (9–11). As a result, a lack of appropriate targeted therapies for breast cancers are classified as triple negative, which are therapies with cytotoxic chemotherapeutic agents, including taxane-based, platin-based, and other DNA damage-causing drugs (12–15).

**Figure 1:**
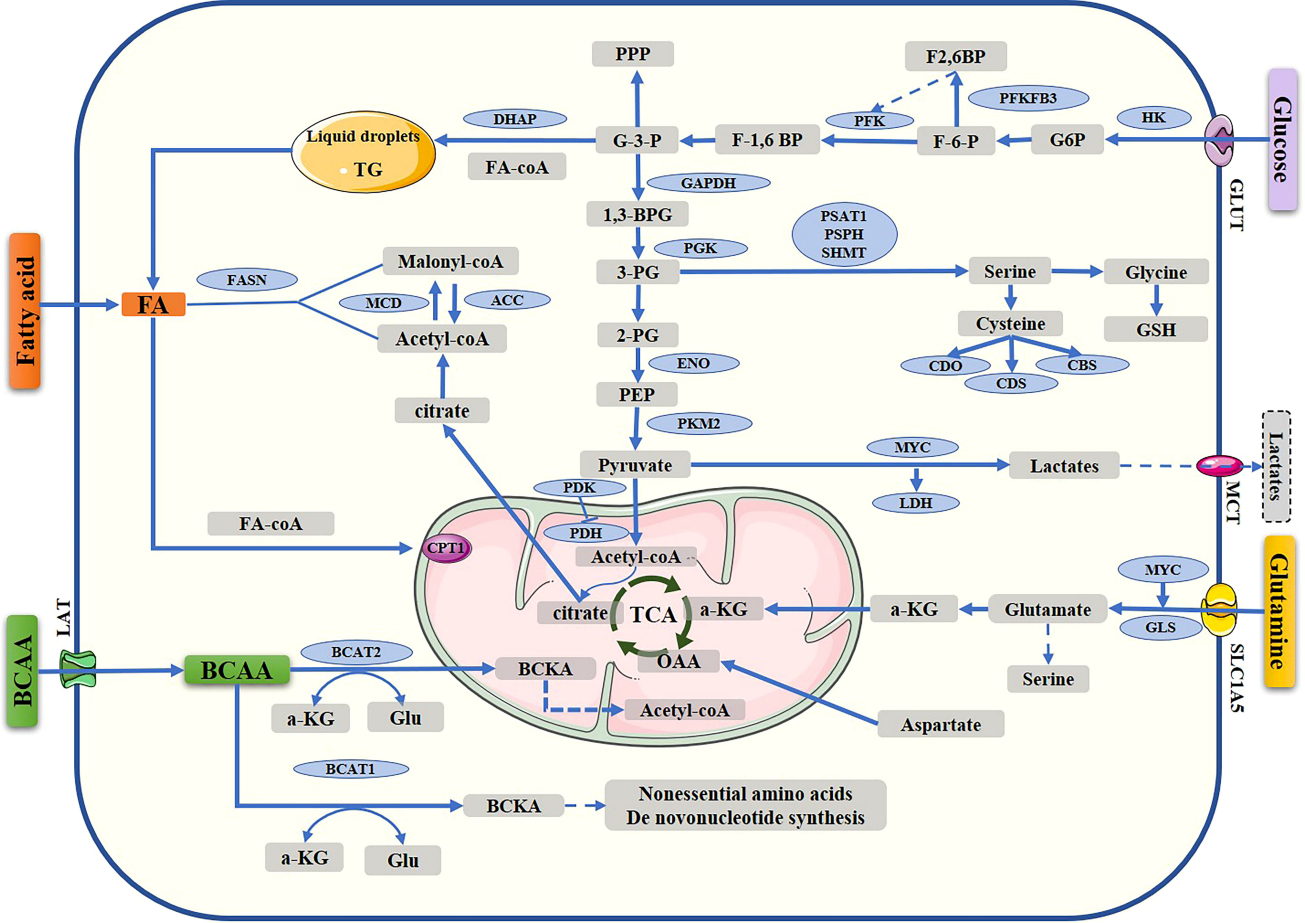
Metabolic pathway in breast cancer cells. Reprogramming of the metabolism, including glucose metabolism, fatty acid synthesis, and amino acid metabolism. TCA, tricarboxylic acid cycle; G-6-P, glucose-6-phosphate; F6P, fructose-6-phosphate; F1,6P, fructose-1,6-bisphosphate; G-3-P, glyceraldehyde 3 phosphate; DHAP, dihydroxyacetone phosphate; 1,3-BPG, 1,3-bisphosphoglycerate, 3-PG, 3-phosphoglycerate; 2-PG, 2-phosphoglycerate; PEP, phosphoenolpyruvate; OAA, oxaloacetate; α-KG, α-ketoglutarate; GLUT, glucose transporter; HK, hexokinase; PKM2, pyruvate kinase isozyme type 2; LDHA, lactate dehydrogenase A; MCT1, monocarboxylate transporter 1; PDK, pyruvate dehydrogenase kinase; PDH, pyruvate dehydrogenase; acetyl-CoA carboxylase; FASN, fatty acid synthase; CPT1, carnitine palmitoyl transferase 1; 3PG, 3-phospho-glycerate; GSH, reduced glutathione; GLU, glutamate; GLUT, glucose transporter; PHGDH, phosphoglycerate dehydrogenase; PSAT1, phosphoserine aminotransferase 1; SLC1A5, solute carrier family 1 member 5; GLS, glutaminase; PSPH, 1-3-phosphoserine phosphatase; BCAAs, branched-chain amino acids; BCAT1, branched-chain amino acid transaminase 1; BCAT2, branched-chain amino acid transaminase 2; BCKA, branched-chain a-keto acid; MCD, malonyl-CoA decarboxylase; ACC, Acetyl-CoA carboxylase; MCT Monocarboxylate transporter.

However, the development of drug resistance reduces treatment effectiveness in breast cancer patients and is an important cause of cancer-related death. Endocrine and HER-2 resistance can result in disappointing outcomes, similar to chemotherapy resistance. Therefore, elucidating the mechanism of drug resistance in breast cancer is crucial to improving rates of survival. It is known that cancer cells possess distinct metabolic properties; among the metabolic properties of cancer cells are increased aerobic glycolysis, fatty acid synthesis, and glutaminolysis (10, 16). (**Figure 1**) Much more attention has recently been paid to targeting metabolic enzymes in cancer therapies and overcoming drug resistance (17, 18). The purpose of this review is to discuss metabolic reprogramming and progress in targeting metabolic pathways to treat breast cancer.

A recent focus in various cancer studies has been the dysregulated metabolism of cancer cells, identifying intratumoral heterogeneity and metabolic abnormalities in cancer cells as likely causes of chemotherapeutic resistance. The various clinical challenges in cancer therapy, such as immunosuppression, inevitable recurrence, anticancer drug resistance, cancer progression and metastasis, also contribute to metabolic abnormalities (19). It has been suggested that the specific metabolic characteristics of tumor cells can overcome the toxic effects of anticancer drugs, possibly leading to drug resistance in tumor cells (20) or promoting lipid synthesis and inducing mutations in such harsh environments (21). Tumor cells may be endowed with a highly adaptive metabolic capacity or benefit from metabolism in the microenvironment and are more likely to evade drug toxicity (22). Overall, metabolic reprogramming is now recognized as a hallmark of cancer. Increasing evidence indicates that metabolic reprogramming is associated with drug resistance in cancer therapy (23).

## The “Warburg effect” and “reverse Warburg effect”

Metabolism of glucose differs significantly between normal and tumor cells. In normally differentiated cells, energy for growth is cells mainly provided by mitochondrial oxidative phosphorylation; in many tumor cells, even when oxygen is sufficient, cells still mainly rely on glycolysis for productivity, which is known as the “Warburg effect”. This phenomenon has been regarded as a phenotype of all cancers (23, 24). The Warburg effect produces substrates that become available for other metabolic pathways, including fat, nucleotide, and amino acid syntheses, that are crucial for oncogenesis (25). In cancer cells, glycolysis is the major metabolic process that produces ATP; the pyruvate formed from glucose must be converted to lactate for it to exert its effects and not be incorporated into the TCA cycle (26). The “reverse Warburg effect” states that cancer-associated fibroblasts can generate lactic acid through aerobic glycolysis, which is then provided *via* a paracrine route to adjacent cells, activating mitochondria, increasing oxidative phosphorylation in adjacent cells and promoting the growth of tumors (27). In general, the “Warburg effect” and “reverse Warburg effect” are both crucial to the development of cancer. Emerging studies suggest that various cancer cell subsets depend on different energy-producing pathways (28).

## Regulation of glucose metabolism in breast cancer drug resistance

The reprogramming of glucose metabolism that occurs in many cancers is to meet the energy requirements of growing rapidly cancer cells (29, 30). Many enzymes play roles in metabolism of glucose, which provides cancer cells with energy. As glycolysis regulators, abnormal expression of glycolytic-related enzymes results in glycolysis dysregulation, which gives rise to oncogenesis, tumor growth, and treatment resistance (31). Researchers have demonstrated the effectiveness of treatments that target metabolism in improving anticancer therapies or reversing drug resistance in breast cancer cells, such as resistance to chemotherapy, endocrine therapy, and HER-2 targeted treatment.

### HK

Hexokinases contribute significantly to the initiation and maintenance of tumors and catalyze the first reaction of glycolysis. This step is a rate-limiting reaction in glycolysis, transforming glucose into glucose 6-phosphate (32, 33). The human hexokinase family consists of three members: HK1, HK2, and HK3 (34). HK2 is highly expressed in many tumors. Some studies indicate that breast cancer cells exhibit a high level of HK2 expression (32). Chemotherapy resistance can also be induced by upregulating HK2 expression, which is an enzyme of crucial importance that is involved in resistance to breast cancer and its prognosis through tumor glycolysis (35).

One study reports that curcumin increases sensitivity to TAM in breast cancer cells by regulating the HK2 pathway. SLUG may also regulate HK2 expression through activation of transcription. Hence, HK2 and TAM resistance may be closely related (36). The mechanism by which HK2 causes TAM resistance was described in depth in another study. When comparing TAMR and MCF-7 cells, TAMR cells exhibit higher HK2 expression and higher glycolysis rates. Both HK2 and mTORC1 are primary sensors of glucose ingestion and metabolism (35). HK2 binds to voltage-dependent anion channels (VDACs) and inhibits apoptosis (37). Additionally, HK2 can be phosphorylated at Thr473, which can cause resistance to paclitaxel (38). Furthermore, dephosphorylation of HK2 at Thr473, SMI 4a resensitizes paclitaxel-resistant cell lines. In preclinical studies, two-deoxyglucose (2-DG), three-bromopyruvate (3-BrPA), and lonidamine (LND) acted as HK2 inhibitors. Trastuzumab resistance correlates with increased glycolysis. It has been demonstrated that trastuzumab combined with 2-DG inhibits glycolysis in breast cancer cells *in vitro* and *in vivo* (39). HK2 knockdown inhibits the proliferation of MDA-MB-231 breast cancer cells and enhances the ability of 5-FU to kill them. When HK2 is downregulated in breast cancer cells, lactate secretion and glycolysis baseline are significantly reduced (40). DZNep, as an indirect inhibitor of histone methyltransferases, potently induces degradation of NSD2 protein and inhibits expression of NSD2 target genes (HK2, G6PD, GLUT1 and TIGAR) involved in the pentose phosphate pathway (PPP). These findings suggest that DZNep-such as agents can be developed to target NSD2 histone methyltransferase for effective treatment of tamoxifen-resistant breast cancer (41). Zhu et al. reported that ETV4, as a pivotal transcription factor, regulates gene expression associated with glycolysis. In the presence of loss of ETV4, glycolytic enzymes, such as HK2 and LDHA, and glucose uptake are inhibited (42). Liu et al. (35) demonstrated that by suppressing the mTOR-S6K signaling pathway, upregulation of HK2 promotes autophagy, subsequently conferring tamoxifen resistance to MCF 7 breast cancer cells.

### PFKFB3

Phosphofructokinase-1 (PFK1) catalyzes conversion of fructose-6 phosphate to fructose-1,6 bisphosphate in the third step of glycolysis. Fructose 2,6-biphosphate, which is produced by the enzyme 6-phosphofructo-2-kinase/fructose2,6-bisphosphatase 3 (PFKFB3) from fructose-6 phosphate, is thought to allosterically activate PFK1. PFKFB3 is expressed at high levels in many cancers (43). PFKFB3 is important for sustaining glycolysis in the tumorigenic environment, even under unfavorable conditions, promoting metabolic reprogramming, cell proliferation, DNA repair, and drug resistance (44). Tamoxifen-resistant LCC9 cells express twofold higher levels of PFKFB3 mRNA and protein than MCF-7 cells. Combining an inhibitor of PFKFB3 with TAM suppresses the growth of both TAMR LCC9 and MCF-7 cells, demonstrating the role of PFKB3 in TAM resistance (45). A combination of PFKFB inhibitors and ER-targeted therapies block tumorsphere formation in several models of advanced breast cancer, such as tamoxifen (TamR)- and paclitaxel (TaxR)-resistant cell models, ER+ patient-derived organoids (PDxO) and murine tumor cells (46).

The glycolysis regulator PFKFB3 is key during BC progression and drug resistance. PIM2 has been identified as a novel binding protein for PFKFB3. PIM2 can directly bind and change the phosphorylation status of PFKFB3 at Ser478 to enhance stability through the ubiquitin−proteasome pathway and to promote glycolysis, BC cell growth, and paclitaxel resistance together with PIM2 *in vitro* and *in vivo* (47). Studies have shown that PFKFB3 stimulation of lactic acid production may mediate activation of the TLR4 signaling pathway to some extent, leading to drug resistance to paclitaxel (48). PFKFB3 is a hub for coordinating the cell cycle and glucose metabolism. PFKFB3 binding results in accumulation of the CDK4 protein by inhibiting ubiquitin proteasome degradation mediated by the heat shock protein 90-Cdc37–CDK4 complex. Proteasome-dependent degradation of CDK4 is accelerated *via* disruption of the interaction of PFKFB3 with CDK4 through lysine 147 to alanine mutation. Blocking the PFKFB3–CDK4 interaction improves the therapeutic effect of the FDA-approved CDK4 inhibitor palbociclib against breast cancer (49). PO is also an inhibitor of PFKFB3, and a study showed that it increases the effectiveness of resistance combined with other anticancer agents (50).

### PK

Pyruvate kinase is a key enzyme that catalyzes the last step in glycolysis. It participates in the process of transferring a phosphate from PEP to pyruvate and converting ADP to ATP. M1 and M2 are the two isoforms of pyruvate kinase, but cancer cells express only the latter. The results of recent studies indicate that many tumors express PKM2, which is a growth factor and an inhibitor of apoptosis. It has a major impact on tumor growth and metabolism (51, 52). Researchers have investigated expression of PKM2 in breast cancer cells, both nuclear and cytoplasmic (53). As a transcription coactivator, PKM2 translocates to the nucleus and increases chemotherapy resistance. In advanced breast cancer, PKM2 expression correlates with cisplatin resistance (54). In MCF-7 breast cancer cells, PKM2 cooperates with sterile 20-like kinase 1 and prevents caspase-3, resulting in inactivation of TAM-induced apoptosis. PKM2 is important in regulating breast cancer cell viability (55). Chemotherapy resistance is also promoted by PKM2 in ER+ breast cancer *via* increased aerobic glycolysis. Accordingly, 2-deoxy-D-glucose (2-DG) is a PKM2 inhibitor that can suppress glycolysis and reverse adriamycin sensitivity in MCF-7 and T47D cells (56).

### ENO

Enolase (EN) is a critical enzyme involved in the Warburg effect. During the phosphorylation reaction, ENO catalyzes conversion of 2-phosphoenolpyruvate (2-PG) to phosphoenolpyruvate (PEP) and ATP. Three ENO isoforms exist: ENO-1, ENO-2, and ENO-3. ENO1 regulates transcription, apoptosis, and cell differentiation. It is also is essential for glycolysis (57). Moreover, analysis of 244 samples of breast cancer tissue revealed strong expression of ENO-1 in ER breast cancer, showing that it is also an important marker for BC (58). In breast cancer, elevated expression of ENO1 has been reported to be closely related to tamoxifen resistance and adriamycin resistance (59), and silencing of ENO expression significantly increases the cytotoxicity of 100 nM tamoxifen in tamoxifen-resistant breast cancer cells. Upregulated ENO-1 suppresses expression of c-Myc, resulting in the survival of resistant cells (60). In MCF7 cells, TAM induces mRNA expression of ENO-1 by activating ERα and NF-κB. As a result, drug-induced apoptosis is inhibited (61, 62).

The correlation between ENO1 and MDR in breast cancer may be regulated by activating the ERK1/2 pathway, and it is likely to be regulated by c-Myc. Therefore, ENO1 alters the concentration of extracellular ATP and further influences tumor cell proliferation (63, 64). Doxorubicin-resistant MCF-7R cells lacks E-cadherin expression and show upregulated Vimentin expression and higher EGFR and ENO-1 levels (65). Furthermore, proteomics profiling studies have indicated that knockdown of ENO-1 expression restores oxidative phosphorylation (66). Above all, an innovative strategy for treating drug-resistant breast cancer is to target EN-1.

### LDHA

Lactate dehydrogenase A (LDHA) is one version of the enzyme LDH. In aerobic glycolysis, LDHA catalyzes the last step of the process, converting pyruvate to lactate, which causes the formation of NAD from NADH. Breast tissue expresses high levels of LDHA, which is one of the most prominent isoforms of LDH (67). According to a recent study, LDHA also plays a significant role in acquired tamoxifen resistance in breast cancer by facilitating autophagy (68). In addition, tamoxifen resistance is associated with changes in LDHA and LDHB gene expression and increased lactate concentrations (69). Thus, LDHA is a great target for controlling TAM resistance in breast cancer. ErbB2 signaling enhances glycolysis *via* LDHA-dependent upregulation of HSF1. Taking a targeted approach to glucose metabolism may help overcome Herceptin resistance in breast cancer. Glycolytic inhibitors combined with chemotherapy overcome resistance and lead to more potent inhibition of glycolysis in ErbB2-positive breast cancer (39).

LDHA correlates with trastuzumab-based therapy resistance (70). LDHA inhibitors suppress proliferation of HER-2-overexpressing cells in breast cancer and increase sensitivity to drug therapy (71). When MDA MB231 cells are subjected to sustained exposure to NAMPT inhibitors, such as FK866, drug resistance is induced based on glycolytic metabolism shifts and LDHA activity (72). Compared to cisplatin alone, electrical pulses (EP) + cisplatin (CsP) cause a switch in metabolism with LDHA downregulation, which impacts TNBC growth, proliferation, invasiveness, chemotherapeutic resistance and poor therapeutic response (73). We observed that LDHA and MCT1 are upregulated in Taxol-resistant breast cancer cells (74). Oxamate, an LDHA inhibitor, combined with paclitaxel induces apoptosis in paclitaxel-resistant breast cells by inhibiting cellular glycolysis. Thus, LDHA may serve as a therapeutic target for breast cancer resistance (75).

### PDC

The pyruvate dehydrogenase complex (PDC) contains three types of enzymes that perform catalytic functions, known as E1, E2 and E3. Cellular metabolic flexibility is provided by the PDC, which integrates glycolysis, fatty acid metabolism, and the TCA cycle (76). PDH is an E1 enzyme that is a component of the PDC that converts pyruvate to acetyl-CoA (77). PDH activity is mainly controlled by pyruvate dehydrogenase kinase (PDK) and pyruvate dehydrogenase phosphatase. By phosphorylating PDH, PDK inhibits PDH activity, whereas pyruvate dehydrogenase phosphatase activates it by reversing phosphorylation. Four different types of PDKs participate in glycolysis. They exert their effects on chemoresistance in tumor therapy and include PDK1-4 (78).

The role of overexpressed PDK in aerobic glycolysis, chemotherapeutic resistance, and metastasis in cancer has been widely studied (79). Researchers have discovered that inhibiting PDK reduces neoplasm development by controlling aerobic glycolysis (80). When PDK1 inhibitors such as triciribine or tetrandrine are combined with tamoxifen, breast cancer becomes more sensitive to the drug (81).

Researchers have shown that hypoxia-inducible factor (HIF)-1α regulates expression of pyruvate dehydrogenase kinase 3 (PDK3), thereby inducing resistance to chemotherapy under hypoxic conditions (82). Additionally, PDK4 alters regulation of PDH and is associated with antiestrogen resistance in breast cancer (83). The pyruvate dehydrogenase kinase (PDK) inhibitor dichloroacetate (DCA) PDK regulates pyruvate dehydrogenase, which aids in the conversion of pyruvate to acetyl-CoA, illustrating the proliferation-inhibiting properties of DCA in highly metastatic diseases (84). By decreasing expression of EGFR, MCF7 cells can be sensitized to tamoxifen-induced apoptosis by DCA (85). In trastuzumab-resistant HER2+ cancers, neuromedin U (NmU) is upregulated, and ectopic expression of NmU increases glycolysis, likely *via* PDK activity, suggesting a possible treatment strategy (86). OSU-03012, which is based on celecoxib as a scaffold to develop a COX-2-inactive PDK-1 inhibitor, potentiates trastuzumab’s antiproliferative effect in HER2-positive cells, especially in SKBR3/IGF-IR cells, through downregulation of PDK-1/Akt signaling (87). By blocking PDK-1/Akt signaling, tamoxifen can be used to sensitize ER-negative breast cancer cells to its antitumor effects (88).

### FBP

Although many previous studies have focused on catabolic glycolysis, recent studies work reveals that Fructose-1,6-bisphosphatase (FBP), as a rate-limiting enzyme that regulates conversion of fructose 1,6-diphosphate to fructose 6-phosphate, is essential for the genesis and development of cancers. Furthermore, the function of FBP in chemoresistance has attracted attention (59). Two types of FBP exist in mammals, FBP1 and FBP2. FBP1 plays a regulatory role in gluconeogenesis, though the physiological role of FBP2 remains unclear. Fructose-bisphosphatase 1 (FBP1) is a target gene of CELF6, and CUG-BP Elav-like family member 6 (CELF6) was identified as an RNA-binding protein. Stable CELF6-overexpressing BT549 and MDA-MB-231 cell lines have been established, and CELF6 overexpression-mediated inhibition of TNBC growth relies on FBP1. CELF6 acts as a tumor suppressor by upregulating FBP1 expression through mRNA stabilization to inhibit TNBC progression and increase sensitivity to PTX treatment (60).

### GLUT

GLUTs are glucose transporters in mammalian cells. The GLUT family comprises 14 members. GLUTs increase uptake of glucose by the cytomembrane and play a critical role in glycolysis (89). Due to oncogenic signaling, it is possible that increased glucose utilization and activated glycolysis, resulting in lactate accumulation, can occur even in cancer cells with oxygen present. Glucose transporter-1 (GLUT-1) expression is higher in TNBC than in non-TNBC (90). Upregulation of GLUT1, GLUT3, and GLUT4 has been related to cancer resistance in several studies. Inhibition of GLUT enhances the anticancer effects of chemotherapy compounds (91). Researchers have recently discovered that GLUT1 plays a role in enhancing autophagy and resistance to TAM in MCF-7 breast cancer cells (92).

Increased GLUT1 transcription and membrane translocation leads to increased glucose uptake and glycolysis through the Akt signaling cascade. Targeting glycolysis *via* Skp2 increases HER2+ tumor sensitivity to trastuzumab treatment (93). Therefore, GLUT inhibitors have been used in a variety of combinations with chemotherapeutics, such as doxorubicin, paclitaxel, and cytarabine, and they exhibit synergistic or additive cancer-fighting effects with reduced chemo-, radio-, and immuno-resistance. Using glucose transporter (GLUT) inhibitors in combination with chemotherapeutic agents reduces chemotherapeutic toxicity compared to monotherapy due to reduced therapeutic doses required to achieve desired effects (94).

In addition, palbociclib in combination with paclitaxel inhibits proliferation of cells and induces apoptosis. By pretreating cells with palbociclib and then removing it before paclitaxel treatment, cell cycle reentry from G1 to S phase can be synchronized. Moreover, palbociclib inhibits glucose transport by reducing GLUT-1 glucose uptake through the Rb/E2F/c-Myc signaling pathway. Furthermore, expression of HIF-1α, a key factor in tumorigenesis, is inhibited. Researchers have shown a high level of GLUT1 in breast and primary colon cancers (95). Phloretin is a GLUT1 inhibitor that inhibits glucose transport and glycolysis. Additionally, phloretin increases the sensitivity of tumor cells to daunorubicin under hypoxic conditions (96). Compound WZB117 has been shown to inhibit GLUT1 in MCF-7 breast cancer cells. There is also evidence of synergistic anticancer effects when WZB117 is combined with cisplatin and paclitaxel. WZB117 inhibits cell proliferation more effectively in combination with a mitochondrial inhibitor, which indicates that it might be more effective against aggressive cancer cells, which are invariably mitochondrial deficient (96). However, combined use of other targeted therapies along with GLUT inhibitors may also be a key strategy for overcoming drug resistance; nevertheless, GLUTs are present in a variety of organs and cells, which makes them difficult to target (97). Consequently, improvement of GLUT inhibitor selectivity and affinity is a major area of study in anticancer research.

Contrary to previous studies, it has been proposed that ablation of GLUT1 attenuates apoptosis and increases drug resistance *via* upregulation of p-Akt/p-GSK-3β (Ser9)/β-catenin/survivin. These results indicate that the potential of Glut1 as a therapeutic target should be carefully re-evaluated (98).

## Regulation of amino acid metabolism in breast cancer drug resistance

Proteins are composed of the amino acids and have structural and functional roles in organisms. Among the various requirements of biosynthesis, amino acid metabolism is vital to maintaining cellular homeostasis, energy production, and redox equilibrium. Furthermore, tumor-specific metabolites, such as polyamines, that play an important role in tumor progression and growth are produced by amino acids (99). Cells resistant to hormonal treatment regulate amino acid anabolism and catabolism to ensure survival and growth. Breast cancer therapeutic resistance is thought to be associated with amino acid metabolites.

### Glutamine

In addition to glucose, glutamine is the most abundant circulating amino acid and functions as a key carbon and energy source for cancer cells. It is known that glutamine is important in cancer because it contributes nitrogen and carbon for a variety of reactions that result in proliferation, invasion, and metastatic spread of cancer cells (100–102). First, by generating α-ketoglutarate (αKG), glutamine serves to provide carbon sources for entry into the TCA cycle. Second, glutamine is an important source of nitrogen for the synthesis of nucleotides and other nonessential amino acids. Last, glutaminolysis-generated glutamate is a precursor of glutathione and helps maintain redox balance (103).

Tamoxifen-induced apoptosis is inhibited by glutamine, and the cooperates between glutamine and stromal cells results in chemoresistance (104). Furthermore, the interaction between the stroma and the epithelium is critical to cancer progression and metastasis. Cancer-associated fibroblast cells produce glutamine, which is then secreted into the tumor *via* autophagy. Glutamine is taken up from the tumor microenvironment and converted to glutamate and ammonia. Upon conversion to α-ketoglutarate, glutamate is used in the TCA cycle, increasing mitochondrial activity. By inhibiting the p53-induced protein TIGAR, glutamine decreases glycolysis, apoptosis, and autophagy (105). Additionally, tumor epithelial cells release ammonia into the microenvironment, where it enters stromal cells, activates autophagy and inhibits Cav-1 expression. Autophagy is proposed to be a common survival mechanism during resistance to TAM (106). Myc is activated in breast cancer cells in the presence of acquired endocrine resistance. In addition to regulating various cell processes, Myc, a proto-oncogene, is involved in glutamine and glucose metabolism (107). Myc inhibition in TAMR cells decreases cell viability, growth, and glucose uptake. Thus, appropriate regulation of the glutaminase-glutamine synthase system (GLS/GAC-GLUL) by Myc is crucial for maintenance of antiestrogen-resistant phenotypes (108). In TAMR cells, endoplasmic reticulum stress is associated with marked upregulation of the unfolded protein response. When glucose is depleted, glutamine induces apoptosis and inhibits autophagy through a pathway mediated by the unfolded protein response (UPR) (109). In breast cancer, c-Myc overexpression may be sufficient to cause antiestrogen resistance (110), and MYC expression is upregulated by crosstalk between ER and HER2 in aromatase inhibitor-resistant breast cancer cells. MYC-mediated glutamine metabolism is associated with AI resistance in breast cancer (111). Re-expression of ERRα in resistant cells triggers metabolic adaptations favoring mitochondrial energy metabolism through increased glutamine metabolism, as well as ROS detoxification required for cell survival under therapeutic stress conditions. Pharmacological inhibition of ERRa activity represents a viable mechanism to counteract lapatinib resistance in breast cancer and to impact metabolic adaptations occurring in resistant tumors (112). It is also notable that the master regulator of mitochondrial metabolism PGC-1a regulates a significant number of pathways implicated in therapy resistance, including OXPHOS (113), oxidative stress response (114), glutamine metabolism (115), and glutathione metabolism (116). The context-dependent roles of PGC-1a may therefore underpin specific metabolic vulnerabilities in both doxorubicin and epirubicin resistance in breast cancer. Targeting global regulators of metabolic plasticity, such as PGC-1a, is promising as a broad strategy for treating therapeutic-resistant cancers (117).

Resistance is common in breast cancer cells, and glutamine addiction is a way to escape drug treatment. As a potential pharmacological target to reverse cancer cell resistance to chemotherapy, glutamine transporters or glutaminolysis have emerged as promising candidates. The amino acid transporter SLC6A14, also called ATB^0,+^, is upregulated in ER-positive breast cancer in women. The features of SLC6A14 include concentrative transport of leucine, glutamine, and arginine. It is possible to inhibit mTOR activity, activate autophagy, and cause cell death by blocking SLC6A14 (118). AI-resistant breast cancer cells show significant upregulation of the glutamine transporters SLC1A5 and GLS, and inhibition of MYC, SLC1A5, and GLS decrease cell proliferation in AI-resistant cells (119). EphA2 is highly expressed in HER2+ tumors, with increased dependence on glutamine metabolism through enhanced transcription of SLC1A5 and GLS, which is recognized as a new target of therapy in HER2+ tumors (120). The glutamine transporter SNAT2 is the AA transporter most frequently induced by hypoxia in breast cancer and is regulated by hypoxia both *in vitro* and *in vivo* in xenografts. SNAT2 induction in MCF7 cells is also regulated by ERα, but it is predominantly a hypoxia-inducible factor 1α (HIF-1α)-dependent gene under hypoxia. A switch in regulation of SNAT2 between ERα and HIF-1α leads to endocrine resistance in hypoxia. The development of drugs targeting SNAT2 may be of value for a subset of hormone-resistant breast cancers (121).

Recent studies have drawn attention to glutaminase, an enzyme that catalyzes glutamine to glutamate and has become a potential target for cancer therapy. A pair of novel glutaminase inhibitors has been found: CB-839 (122, 123) and 968 (124). CB-839 exerts the strongest inhibition of proliferation in TNBC cells but not in ER-positive cells. CB-839 shows significant antitumor activity in xenograft models, whether used alone or in combination with paclitaxel. Compound 968 has the strongest cytotoxic effect against MDA-MB-231 breast cancer cells. Genome analysis indicates that Compound 968 can inhibit apoptosis or promote metastasis gene expression and modify histones. Hence, MDA-MB-231 cells are more likely to be apoptotic and less invasive. When combined with doxorubicin, Compound 968 also increases the chemosensitivity of breast cancer cells.

### Branched-chain amino acids

The branched-chain amino acids leucine, isoleucine, and valine play a pivotal function in tumorigenesis (125). It has been found that breast cancer patients’ plasma and tissues contain higher levels of BCAAs (126). BCAA metabolism enhances the proliferation and growth of breast cancer cells through modulation of mitochondrial biogenesis and function. Catabolism of BCAAs is triggered by the enzyme branched-chain amino acid transaminase 1 (BCAT1). In addition to enhancing citrate synthase activity, BCAT1 increases the quantity of ATP and reduces ROS generation. AMPK, SIRT1, and mTOR are nutritional sensors involved in mitochondrial activity, and experiments have demonstrated that mitochondrial biogenesis is promoted by BCAT1 through its selective mTOR signaling activation. Rapamycin may inhibit BCAT1 by repressing mTOR (126). In TAM-resistant cells, mTORC1 phosphorylates (activates) p70S6 kinase by activating ER signaling pathways independent of estrogen (127). According to one study, leucine uptake is crucial for tamoxifen-resistant cells to grow under nutrient stress conditions. Leucine enters a cell *via* the SLC7A5 transporter (128). The protein LLGL scrawl cell polarity complex component 2 (LLGL2) regulates expression of the SLC7A5 transporter on the cell surface. LLGL2 interacts with SLCA5 and then binds to the YKT6 protein to form a trimeric complex, which results in an increase in transporters on the surface of the cell. Estrogen regulates LLGL2 expression. Overall, expression of SLC7A5 is elevated in TAM-resistant MCF-7 cells (129).

L-type amino acid transporter-1 (LAT1) is involved in chemotherapeutic resistance and may represent a new treatment target in breast cancer. Metabolites of cancer and branched-chain amino acids are also important in energy production and drug resistance in MCF-7 cells treated with chemotherapy, despite reduced glucose metabolism (129).

### Serine

Serine is considered a key factor in glucose metabolism. Indeed, many cancers are associated with upregulation of the serine biosynthesis pathway (130, 131). Glycine is produced by catabolism of serine. Together, serine and glycine provide the primary one-carbon units needed for synthesis of nucleic acids, lipids, proteins, and cofactors (132). Various enzymes are involved in serine metabolism, including phosphoglycerate dehydrogenase (PHGDH), phosphoserine aminotransferase 1 (PSAT1), and 1-3-phosphoserine phosphatase (PSPH), which are highly expressed in TNBC. Additionally, serine and glycine depletion in culture media reduces proliferation of TNBC cells (133).

A variety of cancers highly express 3-phosphoglycerate dehydrogenase (PHGDH), the enzyme responsible for *de novo* serine biosynthesis. In addition to contributing to tumorigenicity, PHGDH may contribute to innate or acquired resistance to current chemotherapies in cancer (134). *In vitro* and *in vivo*, small molecules inhibit the serine synthesis pathway of PHGDH, resulting in a lower proliferation rate of breast cancer cells expressing PHGDH (135, 136). CBR-5884 is a PHGDH inhibitor that suppresses proliferation of PHGDH-dependent TNBC tumor cells (137).

The phosphoserine aminotransferase (PSAT1) gene encodes a key aminotransferase that contributes to serine biosynthesis; 3-phosphohydroxypyruvate is converted to phosphoserine by this enzyme during the oxidation reaction. Transcriptional and immunohistochemical analyses have revealed that ER-positive breast cancer patients who receive TAM are more likely to have a poor prognosis if PSAT1 is overexpressed (138). PSAT1 knockdown sensitizes tamoxifen-resistant MCF7 breast cancer cells to tamoxifen, suggesting that PSAT1 contributes to tamoxifen resistance in MCF7 breast cancer cells. Additionally, combination treatment with YAP/TAZ or PSAT1 siRNA and tamoxifen significantly reduces mTORC1 activity and survivin expression in tamoxifen-resistant MCF7 breast cancer cells. These data suggest that targeting the YAP/TAZ-PSAT1 axis might sensitize tamoxifen-resistant MCF7 breast cancer cells by modulating the mTORC1 survivin pathway (139). In tamoxifen-sensitive MCF-7 cells, overexpression of PSAT1 decreases the inhibition of cell proliferation by 4-OHT. In contrast, silencing either PSAT1 or PHGDH results in a higher response to 4-OHT treatment in tamoxifen-resistant LCC9 cells. Combining a PHGDH inhibitor with 4-OHT also reduces proliferation of LCC9 cells. Overall, these findings suggest that ER+ BC is more likely to develop tamoxifen resistance due to overexpression of serine synthase enzymes. It is capable of being targeted as a novel combinatorial treatment option (140). Studies indicate that kinase inhibitors (KIs) and biguanide agents target various types of cancers in a synergistic and selective manner. The ability of KI/biguanides to effectively treat disease is determined by synthesis of nonessential amino acids (NEAAs). Aspartate, asparagine, and serine synthesis are controlled by the mTORC1/4E-BP axis in response to mRNA translation, and eliminating 4E-BP1/2 significantly reduces breast cancer sensitivity (141).

### Cysteine

TAMR MCF-7 cells have a significantly higher level of cystine metabolism than MCF-7 cells, leading to increased glutathione and taurine synthesis. A higher amount of enzymes related to cysteine consumption is found in the TAMR MCF-7 cells, including methionine adenosyl transferase (MAT), cystathionine b-synthase (CbS), cysteine dioxygenase (CDO), and cysteine sulfinate decarboxylase (CSD). TAMR cells grown in medium lacking sulfur amino acids (SAAD) results in a decrease in cell viability (142). In breast cancer cells, CDO1 restoration leads to increased ROS levels, resulting in reduced viability and growth, as well as anthracycline sensitization. This demonstrates the importance of CDO1 inactivation in breast cancer and its potential as a biomarker and treatment target for overcoming anthracycline resistance (143).

### Aspartate

Autophagy is activated due to depletion of the amino acid pool within drug-resistant cells. To cope with increasing amino acid requirements, TAMR cells promote import of aspartate and glutamate by expressing the SLC1A2 transporter on the cell surface (144). In comparison to normal cells, cancer cells require different amounts of metabolites when they proliferate. Researchers have studied the role of aspartate in cancer. In general, aspartate is necessary for biosynthesis of purine and pyrimidine nucleotides to generate AMP from inosine 5’-monophosphate *via* aspartate (145). The oxidative phosphorylation process provides electron acceptors for aspartate biosynthesis. A lack of electron acceptors prevents proliferative activity in cells. Exogenous aspartate supplementation can increase cell proliferation in cells with insufficient oxidative phosphorylation (146). One study found that ursolic acid increases nuclear accumulation of doxorubicin (Dox) by increasing the amount entering cells and decreasing levels of intracellular alanine, lactate, pyruvate, glucose, α-ketoglutarate, glutamate and various amino acids in the body to reverse MDR. According to the study, UA has potential as an adjuvant antitumor herbal medicine to resensitize cells with chemotherapeutic resistance (143). Consequently, studies have shown that levels of different amino acids and their metabolizing genes determine when treatment ends. Several amino acids are believed to contribute to acquired drug resistance, including serine, cysteine, aspartate, glutamate, and glutamine. Thus, we need to better understand the action of amino acids themselves and their precursors as oncogenic metabolites.

## Regulation of fatty acid metabolism in breast cancer drug resistance

Targeting lipid metabolism is an emerging strategy to enhance the efficacy of anti-HER2 therapies in HER2-positive breast cancer (147). A large amount of lipid and cholesterol is required by cancer cells, which is met by either taking up more exogenous lipids and lipoproteins or promoting *de novo* lipogenesis and cholesterol biosynthesis. Lipid synthesis is crucial to satisfying the anabolic needs of cancer cells (148). As the key enzyme in the fatty acid synthesis pathway, acetyl-CoA carboxylase converts malonyl-CoA to the long-chain fatty acids palmitate and stearic acid (149). Acetyl-CoA carboxylase carboxylates acetyl-CoA to malonyl-CoA. FASN has been found to be upregulated in premalignant lesions as well as in most human cancers. TNBC tumor cells overexpress fatty acid synthase (FASN) (150), and the combination of FASN inhibitors and anti-EGFR signaling agents has significant antitumor effects in preclinical models of TNBC tumors. Overall, FASN activity may play an important role in doxorubicin resistance in TNBC (151).

Blocking FASN inhibits transcription of HER2 by upregulating PEA3, a transcriptional repressor of HER2. Trastuzumab inhibits the HER-2-induced upregulation of FASN expression and fatty synthesis triggered by HER-2 overexpression. In combination with a FASN inhibitor, trastuzumab resensitizes trastuzumab-resistant breast cancers by downregulating HER-2 expression (152, 153). Researchers have demonstrated that FASN regulates HER2 bidirectionally, which should increase sensitivity to trastuzumab (154). Additionally, the FASN inhibitor cerulenin exhibits synergistic effects with docetaxel in HER-2-overexpressing and docetaxel-resistant SK-BR-3 cells, suggesting involvement of FASN in HER-2-induced breast cancer (155). In addition, FASN blockade may promote synergistic chemosensitization of breast cancer cells to other treatments, such as paclitaxel, adriamycin, 5-FU, and vinorelbine (156–159). One study revealed that crosstalk between AKT and AMPK influences autophagy and metabolism (FAO). In turn, AKT activation, autophagy, and FAO are among the mechanisms promoting endoxifen resistance through AMPK (160).

## Regulation of autophagy in breast cancer drug resistance

**Figure 2:**
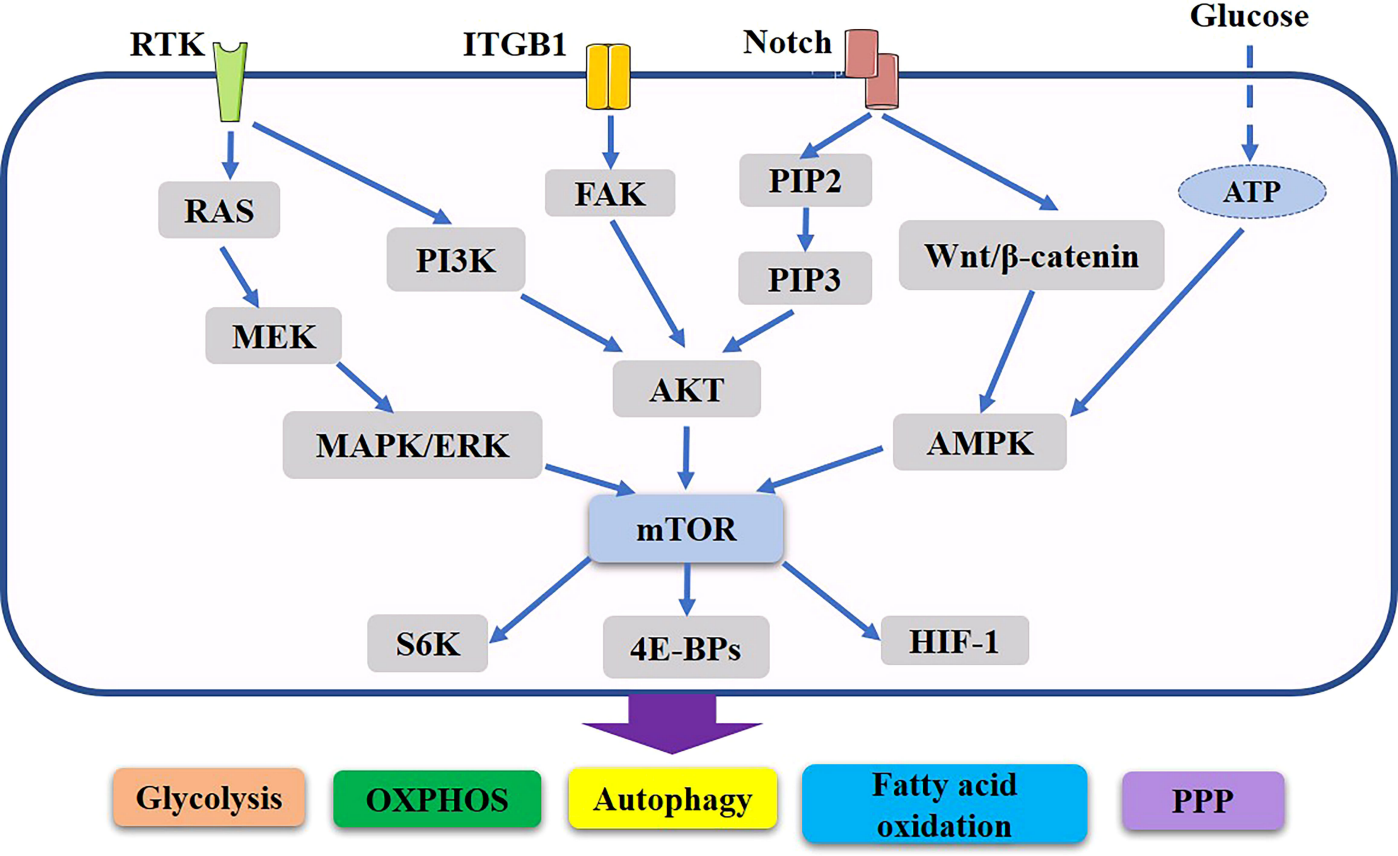
Important role of mTOR related pathway in metabolic reorganization of breast cancer. PI3K and Ras regulate Akt and ERK, which in turn induce changes in intermediate metabolism to promote anabolic processes. Potential Notch signaling crosstalk with other pathways in breast cancer. In addition, they also induce the activation of mTORC1, thus further supporting the rewiring of cellular metabolism and anabolic metabolism progress. Through various mechanisms Akt, ERK and mTORC1 stimulate aerobic glycolysis, lipid synthesis, the pentose phosphate, oxidative phosphorylation, autophagy, thus producing the major components necessary for cell growth and proliferation. These networks of signaling cascades, their interconnection and regulation allow the cells to maintain energetic balance and allow for the physiological adaptation to the ever-changing environment.

During the cancer process, cancer cells experience oxidative stress, which enhances HIF-1α expression and stimulates TGF-α and Caveolin (Cav1) protein loss, downregulating TGF-α; moreover, stromal cells undergo autophagy and become CAFs (161). By undergoing glycolytic metabolism or the Warburg effect, these CAFs provide energy to nearby cancer cells (162). CAFs in the mammary gland are a major component of the tumor microenvironment, greatly contributing to progression of breast cancer. There is a link between drug resistance and enhanced growth, anti-apoptosis, and cell survival processes. However, recent evidence suggests that multidrug resistance in breast cancer cells may also be caused by autophagy (163, 164). (**Figure 2**)

In autophagy, broken organelles such as mitochondria and unfolded proteins are scavenged by autophagy-related proteins, and autophagy-related proteins can also modulate key metabolic enzymes to regulate metabolic reprogramming. Cancer cells can survive by increasing glycolysis when autophagy activity is impaired (165). Researchers have found that autophagy promotes resistance to lapatinib, a HER2/EGFR tyrosine kinase inhibitor, in HER2-positive breast cancer (166) as well as the anti-HER2 monoclonal antibody trastuzumab (167). Enhanced autophagy activity has been demonstrated in doxorubicin- and 5-fluorouracil-resistant TNBC cells (168).

According to a previous study, GPR30-mediated autophagy can reduce apoptosis, thereby conferring resistance to TAMs in breast cancer cells (169). Additionally, CAFs may contribute to TAM-acquired resistance in breast cancer cells *via* the paracrine action of HMGB1, and it has been demonstrated that CAF-expressed GPR30 initiates this interaction. This interaction depends on transcriptional regulation through the GPR30/PI3K/AKT pathway in CAFs and MEK/ERK signaling-induced autophagy in ERα+ breast cancer cells, contributing to TAM resistance (170).

There is evidence that Beclin-1 (BECN1) acts as a suppressor of cancer and is involved in improving autophagy with lysosomal degradation; its expression levels are reduced in mammary carcinomas, particularly TNBC (171, 172). Autophagy-related BECN1 may therefore promote mammary carcinogenesis by negatively regulating metabolic rewiring. As a consequence, loss of BECN1 and autophagy may be linked to metabolic reprogramming and carcinogenesis in TNBC (173).

The proliferation-inducing ligand TNFSF13 (tumor necrosis factor superfamily member 13), which is the ligand for TNFRSF17/BCMA, was identified as an essential gene for B-cell development, autoimmunity, and cancer (174–176). By suppressing the Akt-mTOR pathway, TNFSF13 induces autophagy and therefore desensitizes TNBC cells to chemotherapy drugs such as paclitaxel, doxorubicin, and anthracyclines. Furthermore, TNFSF13-induced autophagy is a useful biomarker for predicting chemotherapeutic efficacy and a potential therapeutic target for reversing chemoresistance in TNBC (177).

In a recent study, it was found that acetylation of lysine 254 (K254) increases activity of GAPDH in response to glucose, which promotes the proliferation of tumor cells (178). The acetyltransferase PCAF and the deacetylase HDAC5 are also involved in reversibly regulating GAPDH acetylation (K254). In addition to increasing glycolysis, GAPDH promotes autophagy of damaged mitochondria, helping to protect cells against caspase-independent cell death (179).

3PO, a PFKFB3 inhibitor, reduces the size of tumors in HER2+ mice with breast cancer (50). One interesting finding (180) is that 3PO treatment-related induction of autophagy provides a mechanism that promotes survival. Consequently, the combination of an autophagy inhibitor and 3PO is recommended to enhance antitumor efficacy. Inhibiting LDHA causes apoptosis and suppresses autophagy in tamoxifen-resistant BC cells, reversing resistance to tamoxifen in MCF-7 and T47D cells (68).

Paclitaxel induction of ER stress in breast cancer cells leads to RNF5 association with and ubiquitination and degradation of SLC1A5/38A2. As a result, Gln uptake decreases, TCA cycle components are reduced, mTOR signaling decreases, and autophagy and cell death are increased (181).

By regulating chaperone-mediated autophagy (CMA), PKM2 K305 acetylation decreases enzyme activity and promotes lysosomal degradation. After acetylation of PKM2, it interacts with HSC70, a chaperone for CMA, and associates with lysosomes. Glycolytic intermediates accumulate in cells expressing the acetylation mimetic mutant K305Q, causing cell proliferation and tumor development. It appears that pyruvate kinase is regulated by lysine acetylation, and the link between lysine acetylation and CMA has been revealed (182).

## Regulation of signaling pathways in breast cancer drug resistance

In tumor cells, increased glucose consumption creates a hypoglycemic microenvironment, and these nutritional deficiencies are regarded by tumor cells as stress signals, which activate the stress signaling pathway to induce autophagy and escape apoptosis. Upstream of the metabolic pathway, several molecules activate the proliferation signaling pathway, promote tumor metabolism, increase glycolysis activity, and inhibit glycolytic enzyme activity while causing drug resistance as a result (25). Several pathways, such as the PI3K/Akt signaling and the Ras/ERK signaling, play a role in anabolic reprogramming (183) (**Figure 2**).

Additionally, MCF-7 cells resistant to tamoxifen exhibit enhanced HK2 and mTOR expression. A mechanism of resistance to tamoxifen occurs by increasing autophagy through inactivation of mTOR-S6K *via* HK2 (35). It was found that drugs with lower mTOR activity were more resistant. Cancer cells maintain aerobic glycolysis and HIF-1α stability despite the absence of hypoxia by the AKT/mTOR pathway or AMPK signaling pathway (184). When inhibition of HK2 suppresses the AKT/mTOR/HIF-1α axis, MCF-7 cells become resensitized to tamoxifen. Through downregulation of EGFR signaling, tamoxifen and dichloroacetate inhibits tamoxifen-resistant MCF-7-cell growth (85). Based on these studies, tamoxifen resistance in breast cancer may be related to AKT/mTOR/AMPK signaling (184, 185).

The PI3K/mTOR, Ras, MAPK and Src pathways are constitutively activated by oncogenic mutations in both normoxia and hypoxia, which increases the level of HIF-1α expression (186). Breast cancer is associated with upregulation of HER-2 levels and activation of PI3K/AKT, which leads to increased stability of HIF-1 *via* mTOR. Blocking the PI3K/Akt/mTOR pathway enhances the radiation response of breast cancer models *in vitro* (187), and phase II clinical trials have shown that CCI-779, an mTOR inhibitor, is an effective treatment for breast cancer (188). Several novel PI3K/Akt inhibitors have been developed in recent years, including SF1126, PI-103, and P529, increasing the effectiveness of radiation therapy and chemotherapy.

It is also believed that FASN is modulated by the PI3k-Akt and MAPK pathways (149, 189). FASN gene expression is increased under hypoxic conditions *via* Akt activation and subsequent SREBP-1 induction (190). In MCF7 cells, MAP kinase inhibition decreases transcription from FASN promoters as well as FASN expression (191). FASN may also be inhibited by rapamycin, an inhibitor of mTOR (192). Recent research indicates that regulation of FASN and HER2 occurs in a bidirectional manner through the HER2-FASN axis (193).

It is known that integrins play multiple functions, including adhesion, migration, and proliferation. They are controlled by the mTOR, HIF-1 and AMPK signaling pathways. In turn, signaling *via* β1-integrin/FAK/PI3K/AKT/mTOR also controls other glucose metabolic pathways (194). Blocking β1-integrin with an antibody before doxorubicin treatment enhances its cytotoxic activity (195). Furthermore, enhanced signaling between fibroblast growth factor (FGF) and fibroblast growth factor receptor (FGFR) is observed in BC cells that are resistant to doxorubicin. Downstream signaling is involved in a variety of oncogenic processes, including angiogenesis, resistance to therapy, and metastasis. FGFR plays a role in increased glycolysis and doxorubicin resistance, according to gene expression microarrays. Furthermore, blocking FGF-FGFR-ERK1/2 signaling with drug inhibitors targeting FGFR4 and ERK1/2 can resensitize drug-resistant phenotypes to adriamycin therapy (196). Although metformin is a hypoglycemic drug, its antiproliferative effects have been demonstrated in various breast cancer cell lines, and it was able to sensitize the multidrug resistance phenotype (197). When metformin is combined with doxorubicin, metformin acts *via* the IFN-α signaling pathway and induces cellular oxidative stress in resistant breast cancer cells, showing higher cytotoxicity than doxorubicin alone (198).

Notch signaling is an evolutionarily conserved pathway. Dysregulation of Notch signaling, for instance, by activating Notch receptor mutations, overexpressing Notch ligands and/or receptors, or overexpressing its target genes, contributes to increased proliferation, cell transformation, and drug resistance in various cancers, including breast cancer, multiple myeloma, prostate cancer, and T-cell acute lymphoblastic leukemia (199). It is known that HER2-driven cancers are aggressive; furthermore, 70% of patients are resistant to targeted treatment (200). Studies have shown that resistance may be caused by direct control of the ERBB2 gene by Notch1 (201), in turn, increased HER2 may activate Notch (202), possibly creating a positive feedback loop. Specifically, trastuzumab-resistant cells express higher levels of NOTCH1, JAG1, and their targets, including HEY1, DTX1, and HES5. However, a decrease in Notch1 expression using siRNA sensitizes these cells to trastuzumab (203).

## Regulation of OXPHOS in breast cancer drug resistance

Great progress in the metabolic reprogramming of tumor cells has occurred in recent decades. A number of molecules act synergistically upstream of the metabolic pathway to regulate the signaling pathway of cell proliferation and increase glycolysis activity and glycolytic enzyme generation and activation, ultimately leading to chemotherapeutic resistance. Hence, drug-resistant cells are endowed with adaptive, proliferative, and survival advantages because of altered metabolism (25). Nevertheless, the notion that resistant tumor cells rely more on mitochondrial OXPHOS and respiration and less on glycolysis challenges the idea that tumors primarily invoke glycolytic metabolism and possess defective mitochondria, as originally proposed by Warburg (204). Metabolic plasticity has been observed in some tumor cells, suggesting a transformation from glycolysis to mitochondrial OXPHOS to produce vast amounts of energy (205).

One study revealed that miRNA-211 controls transcription of PDK4 and that inhibiting PDK4 by miRNA-211 causes BC MDA cells to shift from glycolytic to OXPHOS dominance (77). RNA sequencing has also been applied to analyze differentially expressed genes in tamoxifen-resistant cells. Gene expression patterns suggest dysfunctional mitochondria and translate to OXPHOS (206). Metastatic cancers resistant to hormonal therapies express high levels of CD133 and IL6 and low levels of ER. CD133hi/ERlo also reduces mitochondrial OXPHOS (207). Additionally, two other studies have demonstrate the importance of OXPHOS and highlight the metabolic plasticity of TNBC through enhanced susceptibility to fatty acid oxidation inhibitors (208, 209).

There is growing evidence that OXPHOS participates in tumorigenesis and chemotherapeutic resistance (210). Breast cancer research has shown that OXPHOS supplies most of the ATP required (211). Moreover, OXPHOS influences tumor treatment in a number of ways. Due to the large amount of ATP produced, it stimulates the activity of some transporters, including drug transporters. ABC transporters in breast cancer cells use the ATP produced from OXPHOS to promote efflux of DOX and onset of chemotherapy resistance (205). OXPHOS-induced drug resistance is also associated with tumor stem cells. Mitochondria OXPHOS can cause tumor stem cells to spread and may lead to tumor cell resistance (212). Increasing STAT3 enhances mitochondrial complex I and II activity and thus OXPHOS in mitochondria. Activation of OXPHOS is a mechanism for resistance to TKI treatment (213). In TNBC stem cells, MYC and MCL1 are often overexpressed together, acting as enhancers of mitochondria. They enhance mitochondrial OXPHOS and upregulate HIF-1α expression in synergy; this enhanced mitochondrial OXPHOS promotes BCSC enrichment in TNBC, leading to an increase in chemoresistance. HIF-1α inhibition decreases BCSC enrichment, enhancing chemosensitivity in TNBC cells (212).

Rather than an overly glycolysis-dependent phenotype, recent research suggests that cancer cells can achieve mixed phenotypes of glycolysis and OXPHOS in which ATP production is a result of both glycolysis and oxidative phosphorylation and is critical to supporting the physiological activity of individual cells and thus influencing aggressiveness and therapy resistance (214, 215).

Furthermore, stromal cells interact with cancer cells, promoting tumor metabolism. Stromal cells are induced by cancer cells to invoke aerobic glycolysis, and metabolites accumulated by stromal cells are utilized by cancer cells for the mitochondrial OXPHOS pathway. To more effectively fight cancer, both aerobic glycolysis and mitochondrial metabolism should be targeted (216).

Lactate production and secretion increase as a result of aerobic glycolysis, which eventually results in acidification of the cancer microenvironment. Cancer progression is enhanced by release of lactate into the tumor microenvironment (35).

Monocarboxylate transporter (MCT) is a lactate efflux transporter that is necessary for maintaining pH and regulating glycolysis. MCTs belong to the solute carrier (SLC) family of 14 members (217). MCT-1 is the key element facilitating lactate import, and MCT-4 is a lactate exporter (218). These proteins are present almost ubiquitously in the body, and they are particularly upregulated in cancer cells and CAFs, where lactate is generated and transported. As a result, their overexpression can be used as a biomarker for various types and subtypes of cancer (219). Indeed, there is an association between drug resistance and abnormal expression of the MCT family. For example, MCT1 expression correlates with aggressiveness, recurrence, decreased survival, and tumorigenicity in breast cancer (220). It has been reported that high MCT1 expression causes increases in intratumoral lactic acid, which is associated with poor prognosis (221). MCT1 is a major transporter that assists 3-bromopyruvate (3-BrPA) (222), and MCT1 overexpression in cancer cells increases tumor xenograft sensitivity to 3-BrPA. The study by Morais-Santos et al. found that various subtypes of breast cancer are sensitive to MCT1 inhibitors in different ways (223). A high level of MCT1 expression is observed in TNBC (224). As a direct target of miR-342-3p, MCT1 is increased when miR-342-3p is silenced, enhancing the glycolytic profile of TNBC cells and rendering them more aggressive (225).

In breast cancer lesions, MCT4 is associated with immune cell infiltration, PKM2 and HK3 expression, and glycolytic rate-limiting enzymes. Additionally, MCT4 may play an important role in maintaining the tumor immune microenvironment through metabolic reprogramming. Therefore, these enzymes of the glycolysis pathway (MCT4, PKM2, and HK3) may serve as new targets for modulating the tumor immune microenvironment and enhancing immunotherapy effectiveness (226). MCT4 downregulation overcomes resistance to antiangiogenic therapy (227). Based on studies using xenograft models, MCT4, as a transporter of monocarboxylate across cell membranes, appears to be responsible for secretion of lactate by breast tumor cells. After being secreted, lactate is transported into endothelial cells expressing MCT-1, which triggers the autocrine NF-κB/IL-8 pathway. As a result, lactate signaling induces cell migration and tube formation in endothelial cells, promoting tumor artery morphogenesis and perfusion.

## Conclusion

Cancer research has recently concentrated on the dysregulation of metabolism within cancer cells; metabolic reprogramming is now considered one of the hallmarks of cancer. Increasing evidence suggests that dysregulated cellular metabolism may contribute to drug resistance in cancer patients. According to the Warburg effect, cancer cells invoke glycolysis irrespective of whether they are aerobic or anaerobic, meaning that mitochondrial dysfunction is present (228). Metabolic reprogramming, includes glucose metabolism, fatty acid synthesis, and amino acid metabolism. The fact that metabolic reprogramming occurs in resistant cells and may occur in the majority of tumors has important therapeutic implications and shows that metabolic vulnerabilities might be exploited therapeutically.

In addition, the emergence of the “reverse Warburg effect” indicates that lactic acid serves as a material that provides energy; it can be converted into pyruvate, resulting in stimulated mitochondria and OXPHOS in neighboring cells, and mitochondria are important in many aspects of cellular metabolism (229). Recently, several studies have demonstrated that tumor cells also display metabolic plasticity. When tumor cells are surrounded by ample oxygen or when the external environment changes, glycolysis can moderately transform into OXPHOS. This review, by unveiling key regulatory events, further contributes to our knowledge of the relationship between breast cancer metabolism and drug resistance. To target cancer metabolism in the context of treatment, it is vital to alter the metabolic characteristics of tumorigenesis and the plasticity of cancer cells to switch between different metabolic pathways, survival, and apoptosis inhibition. There are several agents that target specific enzymes in the metabolic pathways of breast cancer, including HK, PK, PDC, GLUTs and lactate, in addition to that targeting metabolism-related molecular pathways and genes in the tumor microenvironment. And potential molecular mechanisms and new methods of treatment have been studied or hypothesized. Several of these agents have been shown to improve the efficacy of current treatments and resensitize resistant cancer cells and have now entered clinical trials. Combining strategies that modulate glycolytic and mitochondrial pathways may be an effective way to eliminate drug-resistant cells.

Overall, proteomic and metabolomic analyses of tumor metabolism provide physicians with insight into therapeutic targets, leading to successful clinical translation. Our hope is that targeting tumor metabolic pathways will play an important role in treating breast cancer in the near future.

## Author contributions

LL reviewed the literature, collected data and wrote the manuscript. SY and YZ revised the manuscript. XZ and SL rechecked the manuscript. XT and DD designed and revised themanuscript. All authors contributed to the article and approved the submitted version.

## Funding

This work was financially supported by grants from the National Natural Science Foundation of China (No. 62072070).

## Conflict of interest

The authors declare that the research was conducted in the absence of any commercial or financial relationships that could be construed as a potential conflict of interest.

## Publisher’s note

All claims expressed in this article are solely those of the authors and do not necessarily represent those of their affiliated organizations, or those of the publisher, the editors and the reviewers. Any product that may be evaluated in this article, or claim that may be made by its manufacturer, is not guaranteed or endorsed by the publisher.
